# Case report: POLE (P286R) mutation in a case of recurrent intestinal leakage and its treatment

**DOI:** 10.3389/fonc.2023.1028179

**Published:** 2023-03-16

**Authors:** Dang Xiang, Gongbo Fu, Yitian Chen, Xiaoyuan Chu

**Affiliations:** ^1^ Department of Medical Oncology, Jinling Hospital, Nanjing Medical University, Nanjing, China; ^2^ Department of Medical Oncology, Affiliated Jinling Hospital, Medical School of Nanjing University, Nanjing, China

**Keywords:** POLE (P286R) mutation, recurrent intestinal leakage, molecular markers, metastatic colorectal cancer, immunotherapy

## Abstract

In recent years, although new drugs and molecular markers have been used to treat metastatic colorectal cancer, there has been little progress in the immunotherapy of advanced colon cancer. The development of sequencing and multiomics technology helps us classify patients more accurately, and then find patients who may benefit from immunotherapy. The development of this advanced technology and immunotherapy based on new targets may herald a new era in the treatment of metastatic colorectal cancer. It is well known that colorectal cancer with dmmr/msi-h phenotype is sensitive to immunotherapy, yet the POLE mutation is the MSS phenotype in colorectal tumors but is also an effective target for immunotherapy. This paper describes a case of recurrent intestinal leakage that required multiple surgical procedures. A high-grade colon adenocarcinoma was identified on surgical histopathology after 18 months, and bevacizumab combined with oxaliplatin and capecitabine proved ineffective against this cancer. An analysis of gene expression indicated that POLE (P286R) mutation, TMB 119.333 mutation per 100 MB, and immune checkpoint inhibitor treatment had a significant impact. This case reminds us that the existence of malignant tumors should be considered for patients with repeated intestinal leakage, and emphasizes the importance of gene detection in the treatment of malignant tumors and the significance of POLE mutations in colorectal cancer.

## Background

Colorectal cancer (CRC) is a malignant tumor with a high mortality rate. Based on global cancer statistics in 2020, CRC was ranked third among cancers and the second leading cause of cancer-related deaths among men and women (1). Advanced CRC Patients remain virtually incurable despite advances in chemotherapy and targeted therapy. The use of immunotherapy has made it possible to curtail disease and achieve long-term disease-free survival.

Intestinal leakage is a rare complication that is primarily associated with inflammatory bowel diseases, intestinal ulcers, intestinal tuberculosis, colorectal cancer, and abdominal surgery. The presence of a fistula in cancer patients indicates an advanced stage of the disease and increases mortality and incidence rates (2). In clinical practice, colorectal fistula is a common symptom of colon cancer. It is easy to misdiagnose early ascending colon tumors because they lack typical clinical manifestations, and the lumen of the ascending colon is large. In the literature, cecal or right colorectal cancer has been implicated as a cause of acute enteritis in 2%–15% of cases (3–5).

In the treatment of advanced cancer, cytotoxic inhibitors of lymphocytic death such as the programmed cell death 1 (PD-1) receptor inhibitor and anti-cytotoxic T-lymphocyte-associated protein 4 (anti-CTLA-4) antibody have revealed impressive effectiveness. Studies have identified several positive predictive markers of immune checkpoint inhibitors ICI, including high-level microsatellite instability (MSI-high), overexpression of programmed cell death-ligand 1 (PD-L1), and elevated tumor mutation burden (TMB) (6). Genes encoding DNA polymerase epsilon (POLE) and delta 1 (POLD1) are crucial for proofreading and fidelity of DNA replication (7). Some case reports have demonstrated a correlation between POLE or POLD1 mutations and the clinical effectiveness of ICI (8, 9).

A POLE-mutated metastatic CRC refractory to both chemotherapy and targeted therapy was treated with a PD1 inhibitor (Terriprizumab) with complete clinical and pathological recovery without toxicity within a relatively short time frame. Advanced colorectal carcinomas are rarely present in this scenario.

## Case report

A 34-year-old male presented with abdominal pain and distension with no previous history of colon cancer. His father died of colon cancer at the age of 50 years and received conservative treatment in the internal medicine department of a local hospital on 2 January, 2019. Due to the symptoms of peritonitis, an exploratory laparotomy was performed on 11 January, 2019, which revealed necrosis of the small intestine (90 cm) and a large amount of bloody fluid and intestinal contents in the abdominal cavity. The final procedure was an ileostomy along with abdominal drainage, which was considered an intestinal fistula. The patient was referred to our hospital’s intestinal fistula specialist on 27 May, 2019. During the colonoscopy, there was widespread polypoid hyperplasia (>100), no neoplastic spaces were found, and the patient underwent two exploratory laparotomies and abdominal irrigations for repeated intestinal leakage in the following year. However, there was no evidence of malignancy in postoperative pathology; however, purulent fluid continuously oozed from the incision. During this period, the patient repeatedly had a high fever of 39°C, and his weight gradually decreased by 25 kg, but he did not vomit blood, show blood in his stool, or suffer jaundice.

**Figure 1:**
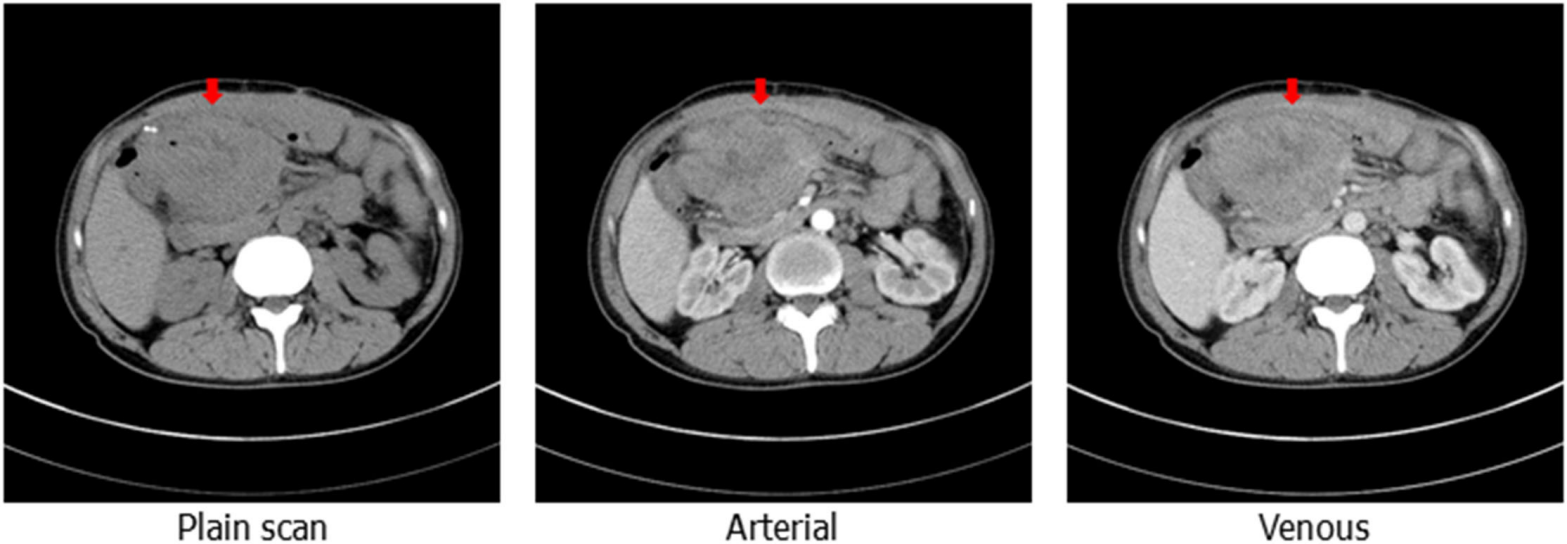
Computed tomography of the abdomen showing the upper abdominal mass with an unclear boundary with the surrounding intestinal canal, obvious enhancement can be observed on enhanced scanning (the red arrow shows the location of the package).

**Figure 2:**
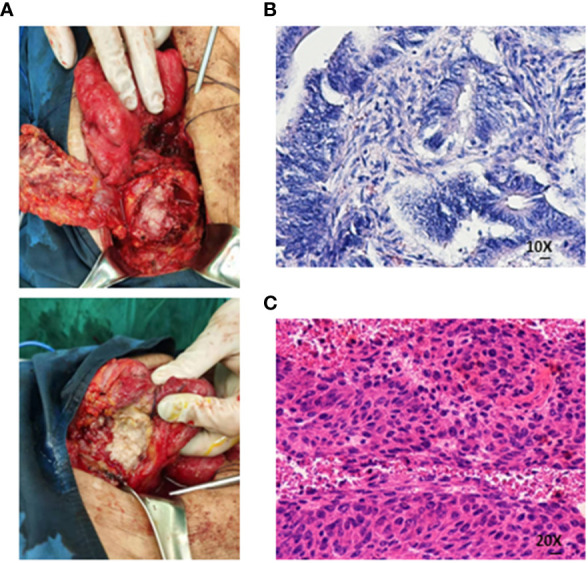
**(A)** The boundary between the mass and the surrounding intestine was unclear, and the central necrosis of the tumor was the flesh of fish-like. **(B, C)** Medium-differentiated adenocarcinoma on histopathological assessment of the resected specimen (Both are hematoxylin-eosin staining).

On 20 May, 2020, a colonoscopy was performed again, and new growths were found around 3/4 of the circumference of the transverse colon wall approximately 60 cm from the anus, which was brittle, hard, and prone to bleeding. Additionally, more than 100 flat polyps with a size of about 0.2–0.5 cm were found in each segment of the colon. Serologically, carcinoembryonic antigen and carbohydrate antigen 19-9 were significantly elevated. The abdominal CT scan depicted an unclear boundary between the middle abdomen and ascending colon, local invasion of the head of the pancreas, and an enhanced scan displayed significant enhancement (**Figure 1**). On 5 June, 2020, the patient underwent a retroperitoneal tumor resection. During the operation, a 0.5×0.5 cm fistula was found at the colon anastomosis, and a 10×10 cm bossing could be touched in the ascending colon and invaded the head of the pancreas, making it difficult to move the fixation (**Figure 2A**). The postoperative pathology illustrated ulcerative moderately differentiated adenocarcinoma with a size of 6 *4.3*3 cm (**Figures 2B, C**). Cancer tissue infiltrated the intestinal wall and was located in the peripancreatic tissue, but no cancer tissue was found in lymph node 0/25.

**Figure 3:**
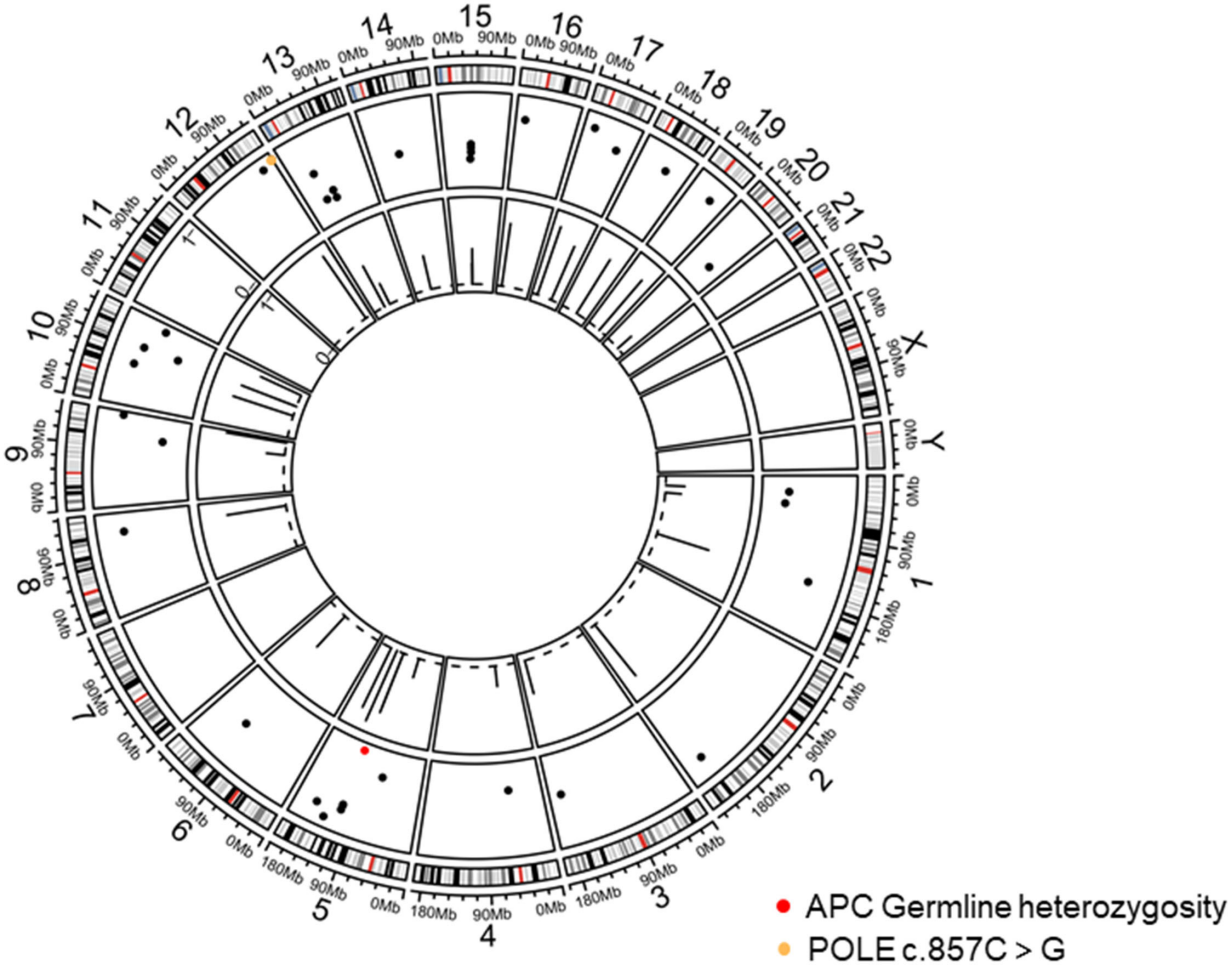
Tumor chromosome copy number map. The red dots display APC germline mutations on chromosome 5 and POLE mutation on chromosome 12.

**Figure 4:**
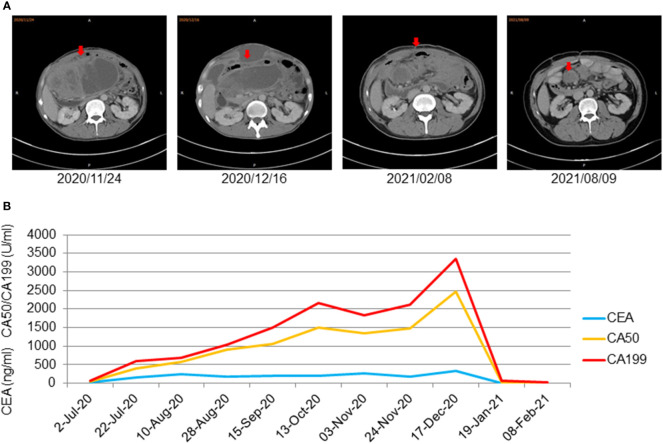
**(A)** CT showed that the abdominal mass decreased gradually after using treprizumab and remained stable for a long time (the red arrow displaysthe package’s location). **(B)** Significant decrease of tumor markers of metastasized cancer under therapy with terriprizumab at 240 mg and bevacizumab at 500 mg every three weeks. Significant decrease of tumor markers of metastasized cancer under therapy with terriprizumab at 240 mg and bevacizumab at 500 mg every 3 weeks.

**Figure 5:**
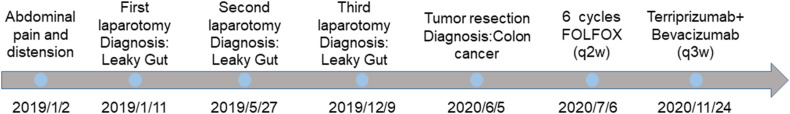
The case lifetime.

On 16 June, 2020, PET/CT scans demonstrated a soft tissue mass (9.7*4.3 cm) in the middle abdomen, considered a malignant tumor. Microsatellite instability (MSI) testing through immunohistochemistry demonstrated that the tumor was microsatellite stable (MSS). Next-generation sequencing (NGS) applying the Illnuina NovaSeq6000 (ACCB Biotech) using the patient’s archival tumor tissue and blood showed RAS wild-type, POLE mutation, tumor mutation load 119.9 mutation/MB (**Figure 3**). A combination of oxaliplatin and fluorouracil chemotherapy was performed every two weeks for six cycles. According to the Response Evaluation Criteria In Solid Tumours (RECIST) criteria, the patient was deemed to have stable disease on CT at weeks 4/8, but the patient was assessed with progressive disease (PD) by CT at week 12. A genetic profile consistent with genetic instability (high tumor mutational burden, POLE mutation) and clear progression of FOLFOX led to the use of terriprizumab and bevacizumab on November 24, 2020. After one treatment cycle, the patient developed a soft abdominal mass protruding from the skin surface. A large amount of purulent fluid was extracted through the puncture and drainage, which healed with repeated irrigation and drainage. After two cycles of treatment, the patient’s disease to was assessed partially responded (PR) by CT (**Figure 4A**), and the carcinoembryonic antigen, carbohydrate antigen 19-9, and carbohydrate antigen 50 levels returned to normal (**Figure 4B**). At the time of this report, the patient had a progression-free survival of 18 months, with ongoing clinical benefits and no immune-mediated toxicities. The case timeline is presented in **Figure 5**. Consent for publication was obtained from the patient for her case report.

## Discussion

Complications of colon cancer can include obstruction, bleeding, and perforation. The clinical manifestation of the disease has been considered a prognostic factor for incidence and mortality (10). Early diagnosis and active surgical treatment may be the key to improving the prognosis of patients. Abscesses have been reported after colorectal cancer perforation. Shang Zhi Han (11) reported on three patients with abscess perforation caused by colon cancer and found that the tumor lesions were located in the right colon. The ascending colon has a large diameter, and the stool tends to be thin. Right colon cancer exhibits fewer symptoms of intestinal obstruction in the early stages than left colon cancer. Colon tumors can mimic abdominal diseases, with a broad spectrum of symptoms. The incidence of colorectal cancer perforation is 2.6%–7.8%, which is a rare case (12).

This case report describes a patient who developed a local abscess perforation. One and a half years after treatment for intestinal leakage, colon cancer was finally diagnosed, making this the most prolonged interval between onset and diagnosis reported in the literature. Additionally, other studies have shown that patients with colorectal tumor perforation have a higher survival rate due to better staging of the lymph nodes (13). This case report revealed a sizeable local mass but no metastases in the surrounding lymph nodes, consistent with the reported literature.

Before diagnosis, no tumors were found in two separate colonoscopy examinations, which is rare in clinical practice. According to studies on the value of colonoscopy, it may lead to interval cancers (ICs) before planned follow-up, accounting for 9% of all CRC diagnoses. The etiology of ICs can be divided into three categories: (1) missed diagnosis, (2) incomplete resection of advanced adenoma, and (3) faster tumor growth rate than that of sporadic colorectal cancer (14). Our case falls into the first category since it seemed premature, and new cancer had appeared, which confirms the experience (15). Willamson B (16) analyzed 39 ICs and found that a third of the 39 ICs had previously been reported to be significantly missed before the recent surgical report after the “difficult test”. Similarly this patient did not continue the first colonoscopy into the intestine due to the large angle at the point where the tube entered the enteric cavity at about 25 cm. This finding is consistent with those reported in the literature. An incomplete exam can lead to missed opportunities, which is also risky. Therefore, the monitoring interval was significantly shortened for those who worked hard and paid meticulous attention to the observation.

Over the past few years, molecular-targeted therapy has become increasingly important in treating advanced colorectal cancer. Keynote 177 trial results depict that pembrolizumab has been approved as a first-line treatment for metastatic colorectal cancer with MSI-high (17). A combination of ipilimumab and nivolumab has also been approved for metastatic colorectal cancer with high MSI or mismatch repair defects. Patients with a POLE gene mutation have been reported to benefit from immunotherapy, suggesting that the POLE gene may be a potential marker for CRC treatment (18, 19).B.J-C. Rousseau et al. (20) reported the first clinical trial to evaluate PD1 in MMR tumors with POLE mutation and found that nivolumab activity appeared in pathogenic mutations of the POLE, such as P286R, V4111L. The POLE mutation site in our case is P286R, which is consistent with the literature, indicating its sensitivity to immunotherapy. The mutation rates of somatic POLE-driving genes are reported to be 2.60% (Zhejiang University cohort), 1.50% (TCGA cohort), 1.00% (Japan cohort), and 1.00% (lancet cohort), respectively. The POLE driver gene mutation significantly increased mutation burden (average TMB of Zhejiang University: 217.98 mut/MB; 203.13 mut/MB in TCGA) (21). The mutation rates of the somatic POLE driver genes are reported to be 2.60% (Zhejiang University cohort), 1.50% (TCGA cohort), 1.00% (Japan cohort), and 1.00% (Lancet cohort). Extreme drive gene mutations significantly increased the mutation burden (average TMB of Zhejiang University: 217.98 mut/MB; 203.13 mut/MB in TCGA) (21). Wang et al. evaluated POLE/POLD1 nonsynonymous variants as biomarkers of immune checkpoint inhibitor treatment outcomes and concluded that they were independent factors for prolonged overall survival after adjustment for microsatellite stability and cancer type (22). It is still unclear why POLE mutations benefit from immune-targeted therapy. Most scholars agree that POLE mutations lead to a high TMB. Mutations in this gene, which contribute to proofreading and fidelity of DNA replication, can lead to a phenotype known as an ultramutator, with a high burden of single-nucleotide variants among human cancers. In many reports, the number of mutations (per Mb) in POLE category tumors is significantly higher than in common hypermutators (23, 24). This category is characterized by a staggering number of mutations and early-onset colorectal or uterine tumors compared to the common hypermutator. In our case, we also found high TMB, probably due to long-term immunotherapy.

The patient’s father died of colon cancer and was diagnosed with multiple adenomatous polyps, no other person in the family has had a similar disease; however, genetic testing was unavailable. Patients with a typical familial adenomatous polyposis (FAP) phenotype have germline APC mutations. In the population without colectomy, 90% of individuals develop CRC during their lifetime. Children older than five years have a higher risk of developing duodenal cancer, pancreatic cancer, medulloblastoma, papillary thyroid cancer, and hepatoblastoma (25). Even though FAP is inherited by autosomal dominant inheritance, more than 30% of patients with APC germline mutations have no family genetic history, and it is speculated that those with index mutations have additional mutations (26). Germline pathogenic variants that affect exonuclease domains (POLE and POLD1) have a high risk of polymerase proofreading-associated polyposis (PPAP). In a national study in the UK which screened 2349 probands, the cumulative incidence of CRC was estimated to be approximately 90% in heterozygotes of the variant pol and 50% in POLD1 (27). NGS revealed the presence of an APC S996fs germline mutation. The mutation was an APC frameshift mutation, and the processing mechanism was evident. Accordingly, it is classified as a possible pathogenic mutation, according to American College of Medical Genetics and Genomics (ACMG). Germline mutations in APC lead to 95% of colorectal cancers (28, 29). Studies have demonstrated that somatic mutations in POLE and PTEN are the main trigger factors of tumorigenesis in tumors with defective POLE proofreading (23), which aligns with the patient’s gene analysis. Patients’ children should undergo next-generation sequencing tests, enteroscopy evaluations, and follow-ups; however, the patient refused because his children were too young.

## Conclusions

In this case report, the clinical presentation and management were unique. The patient was diagnosed with colon cancer after a long time due to inadequate prediction of potential malignancies. Inaccurate diagnosis without recognizing the underlying malignancy can lead to incomplete treatment. Colorectal cancer is a clinically and molecularly heterogeneous disease that requires comprehensive genetic testing to detect rare genetic mutations, such as POLE mutations, to detect tumors harboring an ultramutator phenotype, especially in patients refractory to standard chemotherapy. Further research is warranted to determine whether immuno-oncology therapy could be considered a first-line treatment for patients with cancer harboring POLE mutations.

## Data availability statement

The original contributions presented in the study are included in the article/**Supplementary Material**. Further inquiries can be directed to the corresponding authors.

## Ethics statement

Ethical review and approval was not required for the study on human participants in accordance with the local legislation and institutional requirements. Written informed consent was obtained from the individual(s) for the publication of any potentially identifiable images or data included in this article.

## Author contributions

XC and YC provided the idea of the review. DX and GF wrote the manuscript. All authors read and approved the final manuscript.
